# Harnessing Reliable Evidence in the Post-COVID Era: A Practice Guide to Navigating the Ocean of Medical Literature

**DOI:** 10.7759/cureus.52746

**Published:** 2024-01-22

**Authors:** CheowPeng Ooi, Sivalingam Nalliah

**Affiliations:** 1 Institute for Development, Research and Innovation, International Medical University, Bukit Jalil, MYS; 2 Internal Medicine, International Medical University, Seremban, MYS; 3 Department of Obstetrics and Gynecology, International Medical University, Seremban, MYS

**Keywords:** professional and research ethics, misinformation, patient safety and quality improvement, research methodology, reliability, medical literature, integrity, evidence, disinformation, critical appraisal

## Abstract

The reliability and relevance of medical literature are significant concerns in the post-COVID-19 era, where misinformation and disinformation are serious threats. This practice guide provides an overview of practical strategies to appraise the reliability of research publications critically. These strategies include critically appraising the effectiveness and constraints of various approaches to disseminating medical information, choosing appropriate medical literature resources, navigating library databases, screening the literature from the search, and screening individual publications. We also discuss the importance of considering study limitations and the relevance of the results in research or use in the medical arena. In-depth, critical appraisal of medical or clinical research evidence requires expertise, insight into research methodologies, and a grasp of issues in each field. By harnessing the wealth of reliable and relevant information available in medical literature through the above steps, we can alleviate potentially misleading information and stay at the forefront of our respective fields.

## Introduction

Medical research should provide reliable and relevant findings that can change research and clinical practices. However, the rise of misleading information, disinformation, and misinformation on the internet, social media, and other communication channels in both the public domain and the medical arena has threatened the quality and integrity of medical and scientific literature [1]. Artificial intelligence tools such as OpenAI's ChatGPT, Microsoft's BingGPT, and Google's Bard have recently amplified the disinformation and misinformation tsunami.

Addressing the challenges of misleading information is critical to evidence-based decision-making in the post-COVID era. Reputable medical journals maintain rigorous standards [2]. However, there have been instances of reporting errors, selective data presentation, publication bias, and, in rare cases, publications that deliberately mislead readers. Even rigorous peer review processes assessing research quality, accuracy, and validity before publication may fail to eliminate disinformation. In addition, there are considerations for potential biases and conflicts of interest.

Navigating the deluge of medical publications with the potential to be tainted with disinformation and misinformation in recent years, particularly during the COVID-19 pandemic, requires considerable skill [3]. Unfortunately, many aspiring and current healthcare professionals and researchers lack the advanced skills necessary to assess the reliability and utility of medical evidence [4]. Such inadequate skills may result in ineffective medical decision-making and research planning, leading to the inefficient use of healthcare resources and the avoidance of interventions that may be beneficial or, worse, the use of more harmful than beneficial interventions.

The main goal of this practice guide is to help researchers and aspiring and experienced healthcare professionals improve their ability to critically assess the reliability and relevance of medical literature in the era of disinformation and misinformation. Integrating this comprehensive knowledge into research and clinical practice allows for optimal research findings and outcomes, respectively.

## Technical report

Step 1: critically appraise the different approaches to communicating information on health and medicine

**Figure 1:**
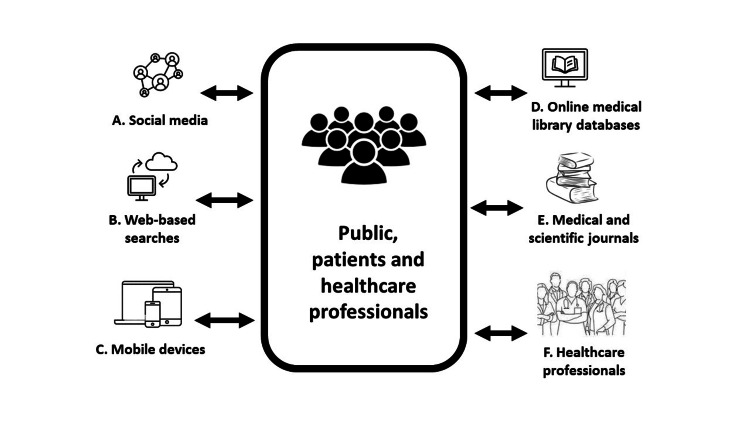
The myriad of approaches to accessing health and medical information

Readers need to critically appraise the effectiveness and constraints of various approaches used to disseminate health and medical information, as illustrated in Figure 1. The emergence of technology and social media has led to various approaches for sharing health information, including evidence derivatives such as plain language or lay summaries, video/graphic/animated abstracts, and audio slides [5]. These methods, accessible in both the public and medical domains, are easier to comprehend but raise doubts about their adequacy for use in research and clinical practice. Thus, health information outside medical journals requires more detail for critical review and decision-making.

Step 2: choose appropriate medical literature resources

To access medical literature, readers should navigate the vast resources available, including web-based MEDLINE, Embase, and PubMed. These comprehensive primary databases index over ten million records from more than five thousand medical and biomedical journals [6]. However, the journals that are indexed in these databases may not overlap. Embase emphasizes drug and chemical nomenclatures and includes conference abstracts that may not be present in other databases. PubMed is an open-access library database, whereas MEDLINE and Embase may require a subscription to access the resources. Since the literature indexed in the different primary medical databases may not overlap, systematically searching all the different databases is required for a comprehensive view of the progress in the respective field. Librarians can help identify relevant resources and provide guidance, enabling readers to use these resources appropriately.

Step 3: navigate to the library databases

**Figure 2:**
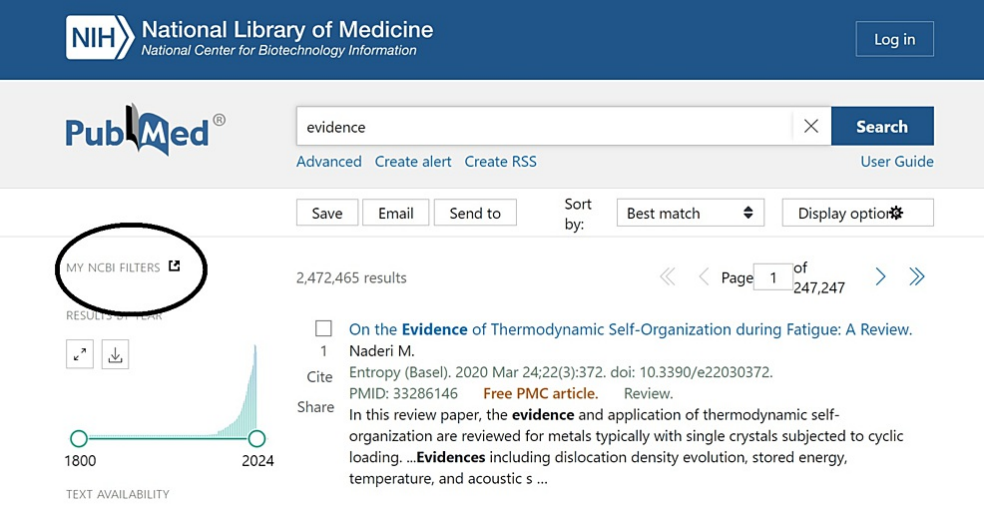
Filters are located on the top left-hand side of the screen

**Figure 3:**
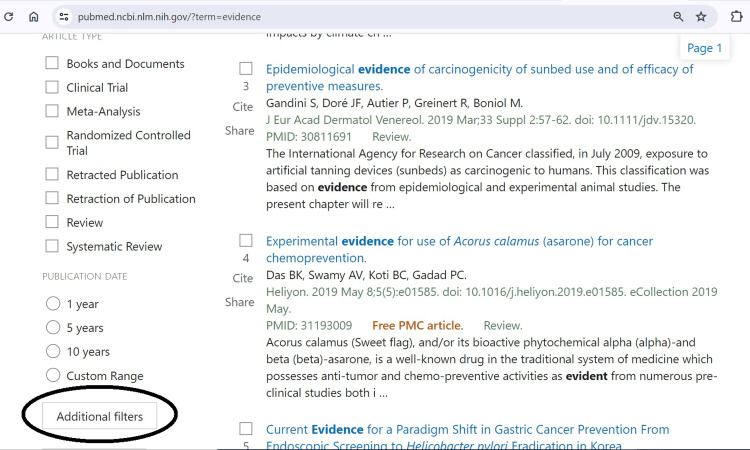
Icon of 'Additional filters' at the bottom of the left-hand side of the screen

We focussed on the PubMed library database of the National Centre for Biotechnology Information (NCBI) platform because it allows open access to all researchers, healthcare professionals, and the public, including those without institutional subscriptions [6]. Users must understand essential search functions, filters, and advanced search features to navigate the medical literature resources. Filters refine the search for original articles, retraction notices, statements of concern, corrected and republished articles, comments, duplicate publications, updates, patient summaries, and retracted articles, as illustrated in Figures 2 and 3. There are also learning guides to navigate the platform. Access to full-text articles is free through PubMed Central or institutional subscriptions.

Step 4: screen the literature from the search results

Readers can subscribe to email alerts from PubMed and other journals to stay updated on new research articles. We suggest using filters within PubMed or medical journal platforms to set up these email alerts for a personalized list of publications. Citation managers, such as Mendeley and Endnotes, can download this list from the email alerts to manage the reference list [7]. These citation tools help format citations, create bibliographies, and share references in various formats.

It is also important to screen for low-quality or suspicious reports from comments in PubMed using the PubPeer extension in the web browser [8]. Readers can counter-check the information in databases such as Retraction Watch and Predatory Reports [8,9]. These databases can help identify and provide details on retracted publications, predatory journals, and publishers. Predatory journals and publishers prioritize self-interest over scholarship, often resulting in misleading information and unethical practices.

Step 5: screen the individual publications

Ethical conduct of research is crucial for maintaining the integrity of scientific inquiry. It includes principles such as honesty, integrity, transparency, and accountability in all aspects of the research process [10]. Research governance encompasses policies, procedures, and regulations that preserve the ethical, scientific, and legal soundness of research studies involving human subjects. It aims to uphold research participants' rights, safety, and well-being while advancing the quality and validity of research outcomes.

Hence, readers need to assess the publications for ethical approvals and concerns about unethical practices during research. Conflict of interest (COI) is a significant concern that can create biases affecting every stage of the research process, from study design to results reporting. Authorship in medical research publications is defined based on the latest recommendations of the International Committee of Medical Editors (ICMJE) and the Committee of Publication Ethics (COPE) [11]. Many journals require listing the roles of the authors who developed the manuscripts. Fulfilling all the stipulated criteria for authorship is essential to qualify as an author. Credibility is critical, and nonhuman entities such as animals, artificial intelligence tools, and bots do not qualify for authorship.

Evaluate the Research Methodology

Scrutinizing the research methodology and process is essential for accurate and reliable results [12,13]. When reading a research article, readers should critically assess and correlate the main research questions and objectives with the study design, including the details of the study design, participant selection criteria, randomization procedures, endpoints, and outcome measures. They should also consider issues that may occur during the research process, such as incomplete recruitment of participants, attrition, deaths, and the interval between the completion of the research and submission of the manuscript. 

The above assessments need to be further complemented by assessing the reporting quality of the article as per the study design recommended by the Enhancing the Quality and Transparency of Health Research (EQUATOR) Network, assessing the risk of bias of the primary studies and the level of evidence using the respective recommended established assessment tools [6,14]. Recommended established assessment tools include the Cochrane Risk of Bias Tool (RoB 2) for randomized controlled trials, the Cochrane Risk of Bias in Non-Randomised Studies of Interventions (ROBINS-I Tool), and the Grading of Recommendations, Assessment, Development and Evaluation (GRADE) approach to the level of evidence [6]. The critical components of evaluating the credibility of the research process are summarised in Table 1. 

**Table 1:** Summary of the critical components for assessing the credibility of the research process

Critical components	Evaluation criteria
Research questions and objectives	Clearly stated and relevant to the research area. Appropriateness in addressing gaps in existing knowledge.
Study design	Clearly described study design (e.g., randomized controlled trials (RCT), cohort study). Appropriateness of study design for research questions.
Participant selection	Clearly defined inclusion and exclusion criteria. Representativeness of the sample to the target population.
Randomization procedures	Adequate description of randomization methods. Minimization of bias through randomization.
Endpoints and outcome measures	Clear identification of primary and secondary endpoints. Appropriateness and validity of outcome measures.
Issues during the research process	Assessment of incomplete participant recruitment. Evaluation of attrition rates and reasons. Considerations of the mortality rate and their impact on the study. Considerations of the time interval between research completion and manuscript submission.
Reporting quality of the research	Evaluation of the adherence to established and recommended reporting guidelines, e.g., the Equator Network database. Evaluation of completeness and transparency in reporting as per established and recommended guidelines.
Risk of bias assessment	Use an appropriate risk-of-bias assessment tool. Evaluation of internal validity of the study.
Level of evidence	Assessment of the strength of evidence of the study using a recommended established assessment tool.

Evaluate the Credibility of Research Findings

Lessons from retracted publications suggest that readers should assess the reliability of the research findings. Authors should be clear and thorough when reporting hypotheses, data collection, and statistical analyses [12,13]. Such things do not always happen. Therefore, it is essential to be vigilant for errors in reporting, incorrect analysis, poor methodology, and any evidence of image manipulations.

Do the published results substantiate the conclusion regarding the impact of treatment on the outcome? Reporting bias is suggested when the data reported does not align with the outcome measures identified in the study methodology or only favorable results are cherry-picked for inclusion. Cherry-picking results often indicate potential vested interests, whether financial or otherwise. 

Readers need to consider whether the data collected are feasible, plausible, or consistent across different groups. Were the statistical methods chosen to show the data in the best light, or could the data be manipulated to show it was statistically significant? Research findings often overemphasize p-values [15]. Thus, considering the causal relationship between treatment and outcome is essential with the increased prevalence of cherry-picked results for their statistical significance. Other common errors include incorrect units, incorrect calculations, and typographical errors.

Be vigilant for publication bias when suggestions favor publishing certain types of research over others. Such publications could indicate a preference for publishing studies that report favorable outcomes, rejecting manuscripts with negative findings, and distorting or misrepresenting results to make them appear more favorable. Allowing the study results to influence journal selection, allowing several journals to publish the results, or publishing the results in different languages are also forms of publication bias. Authors or study sponsors seeking rapid publication, regular or delayed publication, or deciding not to publish the results can skew the evidence on the topic. The key considerations for assessing the credibility of research findings are summarised in Table 2. 

**Table 2:** Summary of the key considerations for assessing the credibility of research findings

Scope of appraisals	Key considerations
Reporting clarity and thoroughness	The authors clearly and thoroughly report hypotheses, data collection, and statistical analyses. Be vigilant for errors in reporting, incorrect analysis, and poor methodology.
Alignment of results with methodology	Verify if published results align with the outcome measures specified in the study methodology. Look for potential reporting bias, especially when only favorable results are highlighted. Be cautious of cherry-picking results, which may indicate vested interests.
Feasibility, plausibility, and consistency	Evaluate if the collected data is feasible, plausible, and consistent across different groups. Assess if the statistical methods chosen may manipulate data to highlight statistical significance.
Causal relationship consideration	Consider the prevalence of overemphasized p-values when examining the causal relationship between treatment and outcome.
Common errors	Be vigilant for incorrect units, calculations, and typographical errors in the published results.

Critically Appraise the Constraints of the Study

Consideration of study limitations is essential when evaluating the results of a study. Study limitations put the results in context, allow readers to critically assess their value, and ensure transparency in the research process [16]. Thus, readers should ponder the study limitations, even though this section may only be a small paragraph or a few lines.

Step 6: apply the results to research or to the practice of medicine

This step is complex. After the above critical appraisal, we suggest that readers further discuss the relevance of the critical appraisal with their senior peers or experts in the field, as well as through journal clubs or research meetings. 

**Figure 4:**
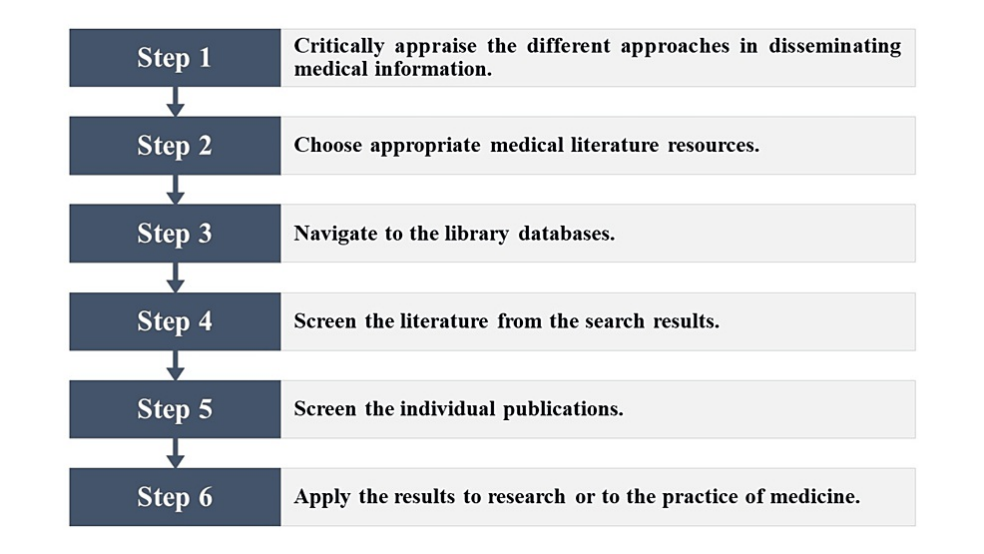
Summary of practice guide to navigate the medical literature

We summarised this practice guide in Figure 4.

## Discussion

This practice guide serves as an overview of practical strategies for assessing the reliability of research publications in the post-COVID-19 era, where disinformation and misinformation in medical literature are significant front-line threats. While disinformation and misinformation existed before the COVID-19 pandemic, disinformation and misinformation intensified and evolved after the post-COVID period [1]. Advancement of technology, particularly the evolvement of artificial intelligence tools, amplified the scale, speed, and impact of disinformation and misinformation during and after the pandemic.

The reliability and relevance of the publication depend on several factors, from the quality of the publication resources to the issues within the individual research and publication. Using established evidence synthesis methodology to synthesize primary research publications may improve the reading experience, especially for those with time constraints. However, harnessing reliable and relevant evidence for use in research and medical arenas requires in-depth and holistic critical appraisal against the background of existing knowledge and professional training rather than mere superficial reading. We recommend that readers correspond with the authors' team to clarify queries that arise during critical appraisal.

The increasing trend of retractions or corrections of published medical literature offers invaluable insights into intentional or unintentional research or publication misconduct [17]. Poor quality data, plagiarism, falsification or fabrication of data, duplicate or redundant publication, ghostwriting, authorship misrepresentation, conflict of interest, ethical violations, and peer review manipulation are common reasons for retracting publications. Author misrepresentation refers to authors misrepresenting themselves or their work [13]. Such misrepresentation includes lying about their credentials, not having the appropriate credentials for the work presented, plagiarising the work of others, and manipulating data to support their arguments.

In evidence synthesis, including fake studies or studies with fabricated data and systematically excluding or overlooking studies with specific findings may distort the representations of the evidence [6]. When there is a bias toward publishing studies with positive or significant results that skew the body of evidence (e.g., publication bias), the synthesized evidence might overestimate the effectiveness of interventions or treatments. Incorporating low-quality studies or overlooking study limitations can introduce flaws in the conclusions drawn from the synthesis of the information. Including studies with selective outcome reporting, where only positive or statistically significant results were published while omitting negative or inconclusive findings, can bias the synthesized evidence. In some fields, such as type 2 diabetes mellitus, the evidence is evolving or complex, with conflicting studies or interpretations [18]. Such fast-evolving evidence scenarios have created much confusion or misinterpretations, even if the research is not intentionally misleading.

Different primary research on the same topic uses different outcomes and outcome measures. Thus, heterogeneous outcomes and measures further hamper evidence synthesis [19]. In addition, misinterpretation or misrepresentation of data within the evidence-synthesis process, including incorrect statistical analyses or misapplication of methods, can also affect the overall findings. Furthermore, through the lens of different experts from different fields, combining studies with conflicting results may yield different interpretations of the synthesized evidence. Finally, researchers must update synthesized evidence frequently in a field where evidence is rapidly evolving, as in COVID-19 during the pandemic [20]. Failure to update can result in outdated conclusions that do not reflect current knowledge on a given topic.

Table 3 summarizes the technical terms used in this practice guide.

**Table 3:** Glossary of technical terms

Technical terms	Definitions
Citation manager	Reference management software for streamlining the process of organizing, storing, and formatting bibliographic citations and references for academic papers, articles, and other research materials, enhancing efficiency and accuracy.
Conflict of interest (COI)	Situations in which persons or institutions doing research may have personal or financial interests that might affect or prejudice their actions or decisions.
Disinformation	Misleading or erroneous information or data that is deliberately spread to deceive or manipulate.
Evidence synthesis	The process of combining data from multiple studies to draw more comprehensive conclusions.
Filter in medical databases	Search criteria for refining search results by focusing on specific study types, publication dates, article types, and other characteristics.
Ghostwriting	A practice where a ghostwriter creates content on someone else's behalf without publicly acknowledging their work, allowing the hirer to present it as their own.
Heterogenous outcomes	Variations in variables or outcomes across multiple research studies on the same topic indicate diversity or variability in variables or outcomes.
Medical library database	An electronic database with a collection of organized and indexed information on biomedical and medical materials, such as research papers, clinical trials, journals, books, and other relevant resources.
Misinformation	A lack of knowledge or misunderstanding frequently causes unintentional dissemination of inaccurate information.
Peer review	A process where the experts in a particular field evaluate and assess the quality, validity, and relevance of research before publication.
Predatory journal	A publication that exploits deceptive or unethical practices, often for financial gain, rather than promoting genuine scholarly communication or scientific knowledge.
Publication bias	A research phenomenon in which the outcome of a study influences its likelihood of publication, with studies producing significant or positive results being more likely to be published.
P-values	A statistical metric used to assess the importance of outcomes in a study.
Risk of bias	Risk of a potential flaw in the design of a study that can distort the relationship between variables, leading to inaccurate conclusions.
Selective outcome reporting	The practice of deliberately presenting only positive or statistically significant results while ignoring unfavorable or inconclusive data.

## Conclusions

Reading an article from a medical journal requires an in-depth critical appraisal of the research integrity, research methodologies, findings, and associated issues to establish the reliability and relevance of the publication. Comprehending the essential issues of intentional or unintentional disinformation in the medical literature that misleads decision-making and causes harm to patients is crucial. In addition, critical appraisal of evidence for medical or clinical research requires expertise, insight into research methodologies, and a grasp of issues within each field. Harnessing the wealth of reliable and relevant information available in medical literature through the above steps may alleviate misleading information and keep researchers and aspiring as well as experienced healthcare practitioners at the forefront of their respective fields.
